# Catarrhal Diseases

**Published:** 1881-04

**Authors:** 


### BOOK REVIEWS AND NOTICES.

CATARRHAL DISEASES OF THE NASAL AND RESPIRATORY ORGANS. By G. N. Brigman, M.D., Grand Rapids, Mich. New York: A. L. Chatterton Publishing Co., 1881.

This little book deserves a place in every homœopathic physician's library, beside the monographs of Bell and King. Catarrhs are often sadly treated by homœopaths; this work renders that difficult task easier. We wish the author had given us a larger repertory; however, that portion can be enlarged in the next edition, which we hope will soon be demanded.

